# Institute of mental health and hospital, Agra: Evolution in 150 years

**DOI:** 10.4103/0019-5545.44759

**Published:** 2008

**Authors:** Sudhir Kumar, Rakesh Kumar

**Affiliations:** Institute of Mental Health and Hospital, Agra 282 002, India

## INTRODUCTION

Lunatic asylums were established by the British in India based on the fact that the care of the insane was the responsibility of the Crown.[1] The first asylum was established in 1745 in Bombay, followed by Calcutta in 1784.[2] There were a few asylums until 1857, which mostly existed in the major cities of Calcutta, Bombay, and Madras. The growth of lunatic asylums was facilitated by the enactment of the Indian Lunatic Asylum Act, 1858.[3] The Act set up guidelines for the establishment of lunatic asylums as well as the procedures for the admission of lunatics. The Agra Asylum came into existence in 1859.[4,5] The circumstances of its establishment were created by the lunacy of the Lt. Governor of Agra Mr. J. R. Colvin who became a lunatic in 1857. In the year of its establishment, the first admitted patient was Aniga, a female beggar who was loitering in the cantonment area. She was admitted on 9^th^ September 1859. In that year, 39 patients were admitted, out of which 25 died, 6 were cured, 7 improved and were declared fit for discharge, and 1 escaped. The administration of the asylum was being managed by the Inspector General of Prisons. Mostly custodial care was provided to inmates in mud houses. The wards and living conditions of inmates were very poor and unhygienic. Lice were seen even on the walls. Many patients died because of diarrhoea.

## A MOVEMENT TOWARDS GROWTH OF PSYCHIATRIC SERVICES: 1904-1956

**Figure 1:**
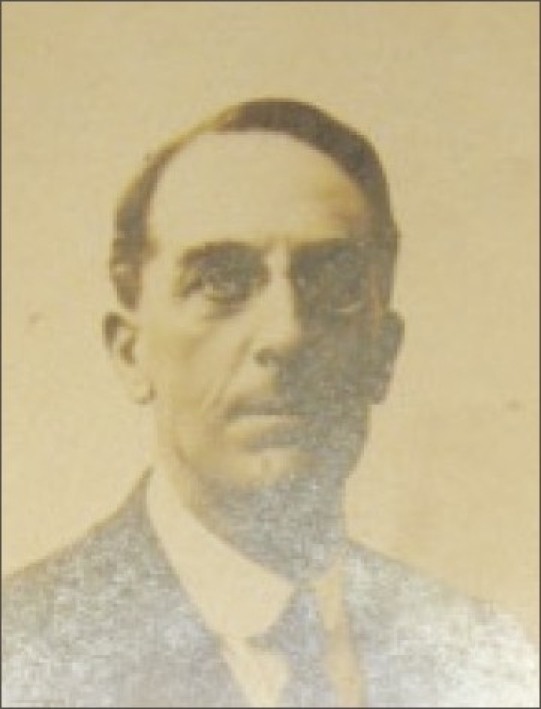
First full time medical superintendent MR. Cochrane (1905-1911)

The Lunatic Asylum in Lucknow was abolished in 1904 and the patients with their case records were transferred to the asylum in Agra. This asylum was granted Provincial Status.[6] The administrative management of the Inspector General of Prisons came to an end in 1905 when Col. A. Cochrane was appointed as the first Medical Superintendent of the asylum [Figure 1]. This marked the beginning of the medical and psychiatric growth of this asylum.

The leadership of the Asylum came into the hands of a leading academician Lt. Col. A.W. Overbeck-Wright in 1911. Apart from his regular services as Medical Superintendent, he served as a visiting lecturer on Mental Diseases to King George's Medical College, Lucknow and Medical School, Agra. He was a member of the Medico-Psychological Association of Great Britain and Ireland. He authored two excellent books in psychiatry: 1) Mental Derangement in India and 2) Lunacy in India.

**Figure 2:**
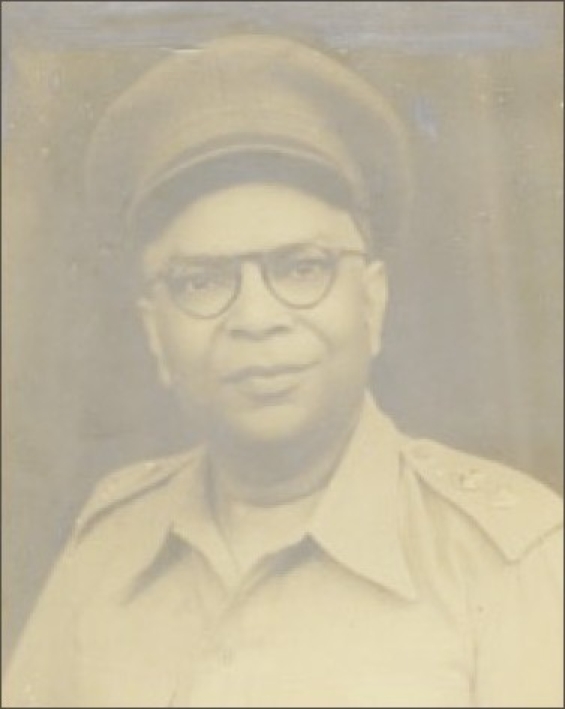
First psychiatrist of Indian origin that headed this hospital, Dr. Banarsi Das (1935-1943)

Like other hospitals, this asylum was renamed as a Mental Hospital, Agra in 1925.[6] Lt. Col. Banarsi Das was appointed as the Medical Superintendent of the hospital in 1934 and served up to 1943 [Figure 2]. His notable contribution was his first real attempt to form an association of psychiatrists in India. He wrote personal letters to the Superintendents of principal mental hospitals throughout the country on June 24, 1935 suggesting that a conference of Indian psychiatrists be held to serve as a ‘forum for the exchange of ideas and act as a clearing house of administrative experience’. He received an encouraging response.[7] However, his attempt was not fruitful until 4 years later, when a meeting actually took place under the auspices of the Indian Division of the Royal Medico-Psychological Association (RMPA) on June 24, 1939. This meeting was attended by 20 psychiatrists under the chairmanship of Lodge-Patch. The second meeting was held in 1941 under the chairmanship of Dr. Banarsi Das. No meetings could be subsequently held during World War II. The Indian Division of the Royal Medico-Psychological Association is considered to be the predecessor of the Indian Psychiatric Society formed in January 1947.[8]

Another highly significant event in the history of this hospital was the commencement of the M.D. course in Psychological Medicine with the initiative of Dr. R.S. Lal who served this hospital from 1943 to 1955 as the Medical Superintendent. The first M.D. thesis was submitted by Dr. S.C. Srivastava under his guidance and supervision in 1955 on the topic ‘Epilepsy: Abnormal behavior and crimes associated with it’. It is worth mentioning here that the 6th Annual Conference of the Indian Psychiatric Society was held in Agra on January 5–7, 1953.

## THE GOLDEN PERIOD: 1957–1975

**Figure 3:**
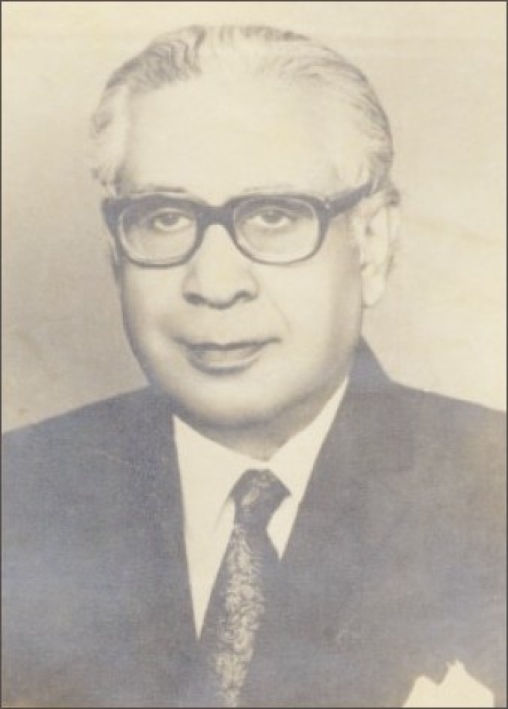
Architect of the Golden Period: Dr. K.C. Dube

The Mental Hospital, Agra reached its peak during the period of 1957–1975 under the dynamic leadership of Dr. K.C. Dube [Figure 3]. Within a year of his joining, he took a bold step and unlocked the psychiatric wards of the hospital. He developed the idea of an open door policy independently in this hospital on the basis of experiencing much difficulty in the management of the wards with locked doors. There was great apprehension and panic in the staff especially the attendants and head attendants who were in charge of the patients. The main point of worry was that there would be an increase in escape and violent attacks. Gradually, these doubts and apprehensions dwindled away giving place to tranquility and relief.[9]

**Figure 4:**
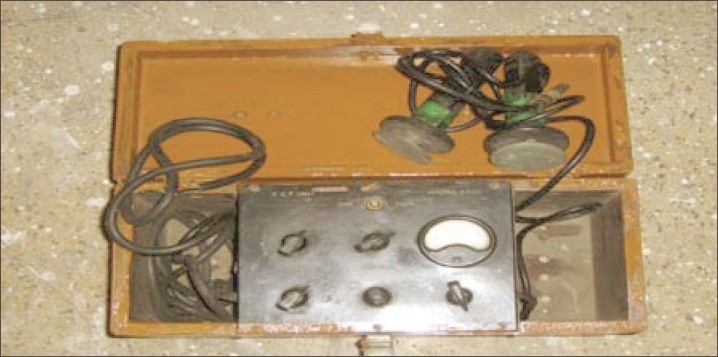
ECT machine

**Figure 5:**
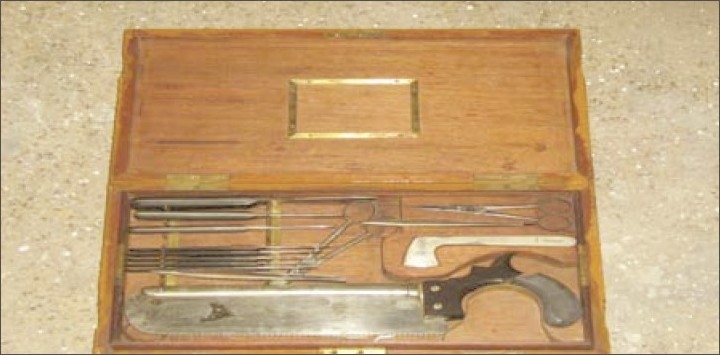
Instruments for transorbital leucotomy which were being used in this hospital

**Figure 6:**
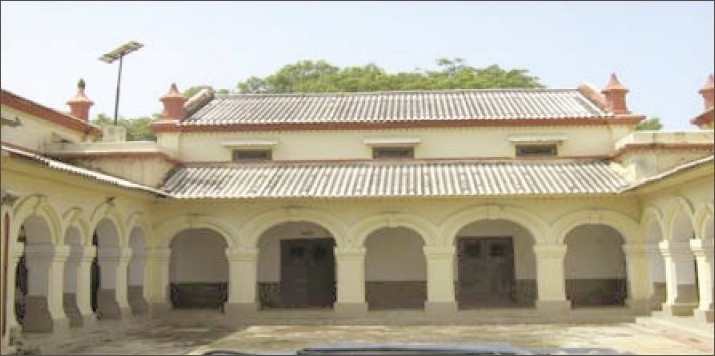
Old infirmary ward

**Figure 7:**
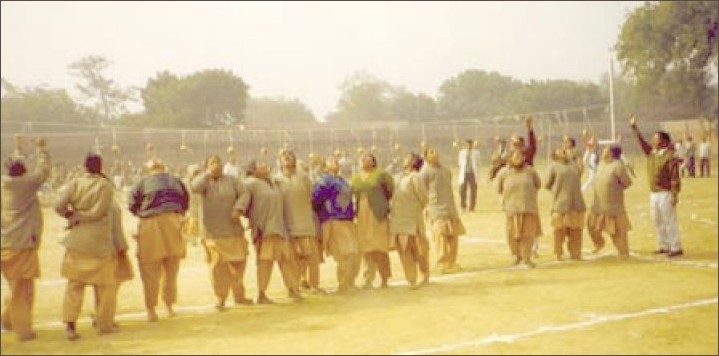
Female patients taking part in apple race on 26th January 2008 – Annual Sports Day

**Figure 8:**
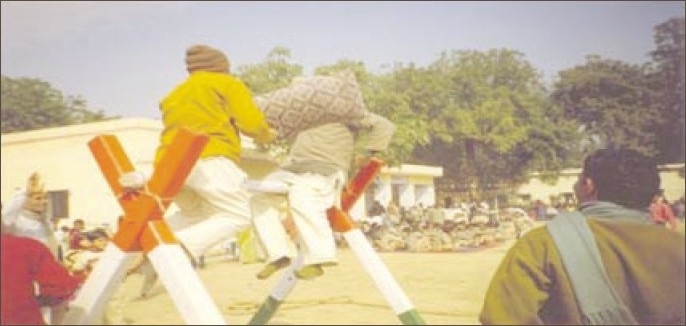
Male patients taking part in pillow fight on 26th January 2008 – Annual Sports Day

In those days, insulin treatment was used quite frequently in the classic manner. It is interesting to note that modified electroconvulsive therapy (ECT) [Figure 4] was also practiced in suitable cases as a routine through the use of relaxants, Brevedil, and Scoline. The Glissando method was also in use here. This hospital was one of the first two centers in India where this treatment was available. Transorbital Leucotomies [Figure 5] were also being performed in selected cases. Equipment for psychosurgery such as a hydraulic operation table, electrocautery, and Boyle's anesthesia apparatus were available. A separate ward was dedicated to physically sick patients [Figure 6]. By 1960, malaria had become almost non existent. Diarrhea and dystentry had reduced to a negligible minimum.[10] For effective rehabilitation of the patients, an innovative Kaman system was in force. In the Kaman System, the patients were deployed in small groups to participate in various hospital-based activities like agriculture, campus maintenance, etc. The system still continues today. There continues to be a provision of annual sports held on 26^th^ January each year in which patients and staff members participate in many sports events [Figures 7 and 8]. This activity is very popular among patients and staff alike. Also, the maintenance of every ward is assessed by a team and a shield is given to the best maintained ward.

An important event in the history of psychiatry in India occurred in 1960. In this year, the Health Ministry of the Government of India convened a 2-day conference of the Superintendents of Mental Hospitals of India to draft a revision of the age old Indian Lunacy Act of 1912. The conference was organized at this hospital on November 25-26, 1960. Around the same time, the Indian Medical Council had appointed a sub-committee to formulate curriculum for postgraduate training in psychiatry and appointed Dr. K.C. Dube as convenor of the committee.[11]

The hospital had a distinct research contribution. A number of Indian Council of Medical Research (ICMR)-sponsored research projects had been executed. The most important being ‘A pilot investigation of the incidence of mental diseases in India (1961–1967)’. This has so far been one of the largest epidemiological studies in India and is often quoted in research publications globally.[12] The hospital hosted the 15^th^ Annual National Conference of the Indian Psychiatric Society in March 1962.

In 1966, the Mental Health Unit of the World Health Organization (WHO) selected the Mental Hospital, Agra as one of the nine field research centers for their prestigious research project “The International Pilot Study of Schizophrenia”. The other eight centers were located at Aarhus (Denmark), Cali (Columbia), Ibadan (Nigeria), London (UK), Moscow (USSR), Prague (Czecoslovakia), Taipei (Taiwan), and Washington (USA). The report was published by WHO in two volumes (1973 and 1979). Dr. K.C. Dube had been a Member of the WHO's Expert Advisory panel on Mental Health (1966–1977). More than 100 research papers were published in reputed National and International Journals.

## THE DULL PERIOD: 1976–1993

The trend set by the Golden period continued for about one decade under the stewardship of Dr. B.S. Yadav. Gradually, there was a decline in growth-oriented activities in the hospital. The National print media published some adverse reports in the context of this hospital, which led to the Public Interest Writ Petition in the Honorable Supreme Court of India. Before that, a Writ Petition was in progress for Ranchi Mansik Arogyashala in the Apex Court vide writ petition no.339 of 1986. The case of Mental Hospital, Agra was added to the petition. After a detailed work up, the Honorable Court drafted and directed a petition for major changes in the structure, objectives, and functioning of the Mental Hospital, Agra in its judgment of September 1994. The status as a State-owned hospital was converted into an autonomous Institution. The Mental Hospital, Agra was renamed as Agra Mansik Arogyashala.

## THE ERA OF AUTONOMOUS INSTITUTION: 1994 ONWARDS

The Government of UP declared Mental Hospital, Agra as an autonomous institution in 1995 and renamed it as Institute of Mental Health and Hospital, Agra in 2001 to promote the development of teaching, training, and research activities. The Honorable Supreme Court specified the following objectives:

1. To provide diagnostic and therapeutic facilities for mental patients
2. To develop an infrastructure for providing social and occupational rehabilitation to mental patients
3. To provide professional and para-professional training in the fields of Psychiatry, Clinical Psychology, Psychiatric Social Work, and Psychiatric Nursing
4. To extend Mental Health Services at the community level by providing training to medical and para-medical personnel in the field
5. To conduct research in Behavioral Sciences

**Figure 9:**
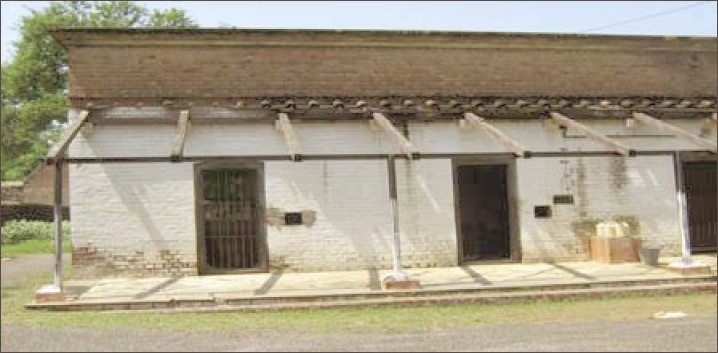
Dismantled single cells

**Figure 10:**
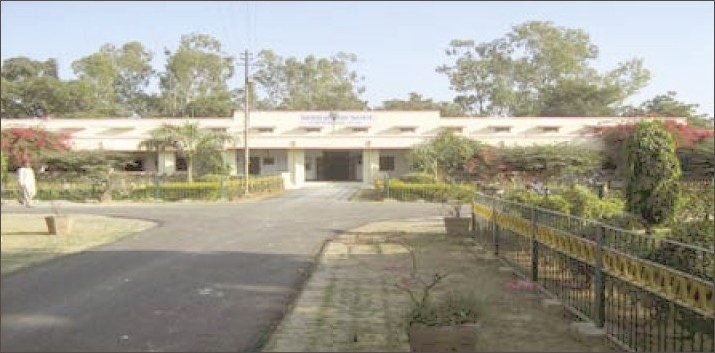
Current administrative block of the Institute

**Figure 11:**
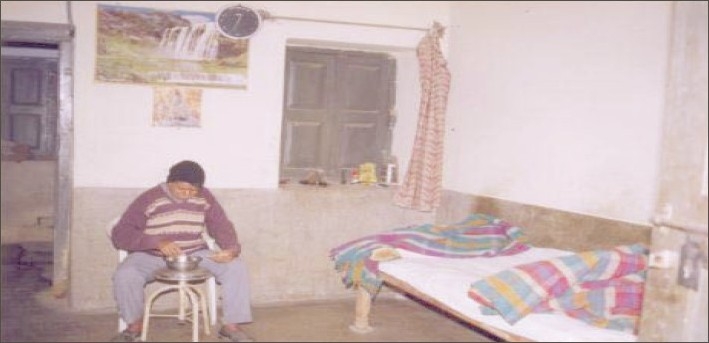
A patient in his half-way home room

**Figure 12:**
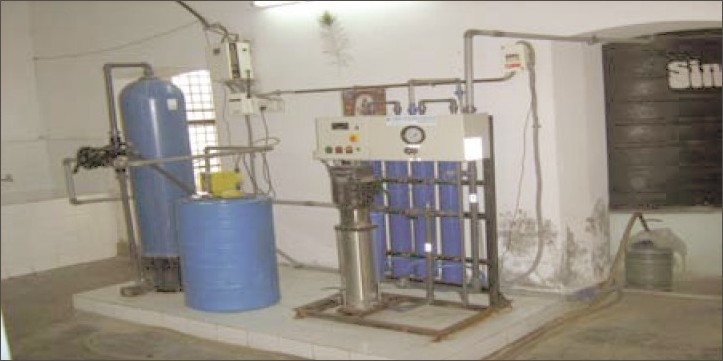
R.O. plant from which safe drinking water is supplied to the patients

Having attained autonomous status, the Institute has seen multidimensional growth in almost all areas under the directorship of Prof. Sudhir Kumar. The Institute is spread out over a land area of 172 acres. The intake capacity for indoor patients is 718. To promote awareness and rapid management of psychiatric patients, the concept of a Family Ward has been introduced. A beautiful park has been developed within the premise of the family ward. A patient stays with at least one of his family members in this ward. The average duration of stay is about 10 days. If there is sufficient improvement in the condition of the patient, he is discharged with follow-up advice; otherwise, the patient is transferred to the general ward. The old single-cell system for the isolation and restraint of patients has been dismantled completely [Figure 9]. The Administrative Office shifted from the old block to the New one [Figure 10]. A Half-way Home with an intake capacity of 10 patients has been established where patients are allocated separate rooms with unrestricted access to various areas of the hospital. The patients who have improved considerably and need reintegration into society are transferred to the Half-way home and are engaged in meaningful hospital activities [Figure 11]. Earlier, there was no provision for catering to the needs of psychiatric emergencies. An Emergency Ward has been established. The Recreation Unit has been augmented with many indoor and outdoor games and basic gym equipment. Besides modern treatment facilities, computerized systems have been installed for a modified ECT, Biochemistry Laboratory, and Psychodiagnostic Unit. Many modern gadgets and appliances have been installed in the hospital to ensure maximum care and ease for the patients. These include the following: a gas-based centralized kitchen, reverse osmosis (RO) system to provide safe and clean drinking water [Figure 12], incinerator with computerized autoclave, shredder and chemical treatment tank to take care of biomedical wastes as per World Health Organization (WHO) norms, and a digital Electronic Private Automatic Branch Exchange (EPABX) system. Computerized electroencephalograms (EEGs), electrocardiograms (ECGs), and ventilators have also been procured.

By now, most of the long-stay patients have been discharged. Community Mental Health services are being provided through satellite clinics at Ram Krishna Mission, Vrindavan, and other clinics in adjoining districts of Agra. Regular short-term training programs are conducted for medical, psychology and social work and nursing students from various parts of the country.

**Figure 13:**
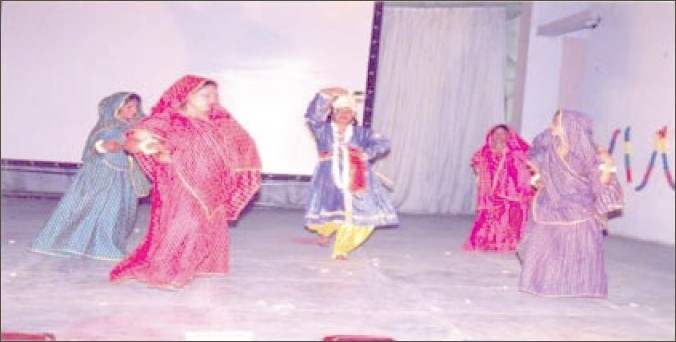
Female patients presenting a group dance in the auditorium – Holi Celebration

There is an auditorium with a sitting capacity of 500 people. Periodically, cultural programs are organized in the auditorium for the recreation of the patients. Most of the time, joint live performances are given by the patients, students, or invited cultural groups [Figure 13]. Every fortnight, educative and entertaining movies are shown to the patients through an LCD projector. The patients take keen interest in the programs. The agricultural work has been reintroduced for occupational engagements of the patients. Occupational Therapy units are functioning for both male and female patients separately. Many new trades have been introduced. A system of remuneration to the patients engaged in occupational activities has been introduced in this hospital. Depending upon their output, a sum of Rs. 25–30/per day is credited in the personal account of the patients as an incentive for their rehabilitation/work activities. Previously, there was no provision for bathrooms and dinning halls for the patients. Now, each ward complex has been provided with bathrooms and dinning halls to bathe and eat with dignity.

The Institute is subscribing to 21 national and 12 international journals since 1999. A subscription to e-journals through EBSCOhost.com has also been added, which provides online access to 540 reputed international journals. Important textbooks and reference books in psychiatry and allied disciplines have been procured in the library of the Institute.

**Figure 14:**
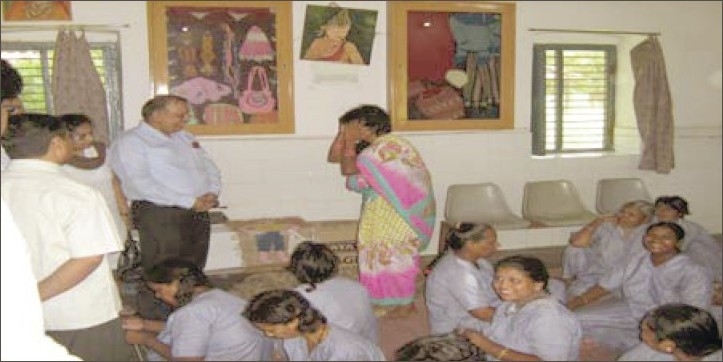
Shri Akhil Kumar Jain, Secretary General, National Human Rights Commission, New Delhi interacting with female patients working in occupational therapy unit

A separate research division has been established in the Institute. It has been recognized by the Department of Scientific and Industrial Research, Ministry of Technology, New Delhi. About 50 research papers have been presented/published in National and International Conferences/journals. The Indian Council of Medical Research has sanctioned an adhoc research project titled “Effects of remunerative jobs on psychopathology and psychosocial dysfunction in hospitalized chronic schizophrenic patients”. A total of 33 research projects have been completed by postgraduate students of psychology in the Institute. Dr. B.R. Ambedkar University, Agra is in the process of finalizing the affiliation formalities for professional postgraduate courses in psychiatry, clinical psychology, psychiatric social work, and psychiatric nursing. DNB in Psychiatry has commenced in 2008. The National Human Rights Commission monitors the progress of this Institute regularly [Figure 14]. The Institute is proud to be the host for 61^st^ Annual National Conference of Indian Psychiatric Society, (ANCIPS) 2009.

## CONCLUSION

The Institute of Mental Health and Hospital, Agra is a famous and premier center of treatment, care, rehabilitation, teaching, and training in the country. It has seen both ups and downs since its establishment. Now is a period of renaissance for the Institute. The National Human Rights Commission, Union Government, and the Government of UP have shown their initiative to raise it to the level of a Center of Excellence in Mental Health.
